# A Narrow Chance

**Published:** 1891-09

**Authors:** 


### A NARROW CHANCE.

William Sauer, a young law student, had a recent experience which he will never forget. Bright and early in the morning he started out for a good, long bicycle ride, heading toward Montgomery, a place in Hennepin county, seven or eight miles beyond Fort Snelling. He reached the village, and, after a good rest, turned about and started back. Three miles on the way he became thirsty, and discovering a well at an unoccupied farm house, halted to get a drink. He stepped on to the boards which surrounded the pump and commenced pumping. He had not made more than three strokes when the board upon which he stood broke in two, and he dropped to the bottom of the well, a distance of fifty feet. He was stunned, and remained in a dazed condition for some time. When he at length came to his senses he began calling for help. The well was back from the road at least 150 feet, and there was not a residence within a mile of the place. After a half hour of long effort his eyes were sufficiently used to the darkness of the hole to enable him to see a little. A small pipe extended down into the water from the pump above.

He wrapped his arms and legs about it with the hope of being able to climb it. It was damp and slippery, and when he got up two or three feet he slipped back into the water. He tried it over again with the same result. Then he tried calling again. He was finally so hoarse that he could not utter a sound. He made another desperate effort to climb the pipe, but it was a failure. Then he gave up hope. But as one thing and another trooped through his mind it seemed to him that he was too young to die. There was too much in life he had hoped for. The thought was too terrible. He became very calm and canvassed the situation carefully. If he could climb the pipe ten or twelve feet he would reach the point where the well hole became so narrow that he could touch the stone wall with his feet. He mustered all his strength and wrapped himself about the pipe once more. Again and again he climbed part way up and slipped back. Once he was so far up that he could almost touch the wall with a hand. And then ! down into the water he went again. At length an idea struck him. He took off his shirt, tore it into strips, dug up sand from the bottom of the well and worked it into the cloth, and wrapped the strips around the pipe as far up as he could reach. Then he climbed up and wrapped strips still farther up. At last with one superhuman effort he planted a foot on a stone in the wall. After a few moments pause to catch his breath he began moving upward, and very soon he was on the outside once more. He was badly bruised, but not seriously hurt.

